# RY10-4 Inhibits the Proliferation of Human Hepatocellular Cancer HepG2 Cells by Inducing Apoptosis *In Vitro* and *In Vivo*

**DOI:** 10.1371/journal.pone.0151679

**Published:** 2016-03-14

**Authors:** Xuenong Zhang, Yanyan Wang, Shishi Han, Huiyao Xiang, Yan Peng, Yinghua Wu, Songwei Pan, Ye Zhang, Jinlan Ruan

**Affiliations:** 1 Department of Pharmacy, The First College of Clinical Medical Science, China Three Gorges University & Yichang Central People’s Hospital, Yichang, 443003, China; 2 School of Pharmacy, Tongji Medical College, Huazhong University of Science and Technology, Wuhan, 430030, China; 3 Department of Clinical Laboratory, The First College of Clinical Medical Science, China Three Gorges University & Yichang Central People’s Hospital, Yichang, 443003, China; University of North Carolina School of Medicine, UNITED STATES

## Abstract

This study aimed to investigate the anti-tumor activity of RY10-4, a small molecular that was designed and synthesized based on the structure of protoapigenone. A previous screening study showed that RY10-4 possessed anti-proliferative effects against HepG2 human hepatocellular carcinoma cells. However, the full range of RY10-4 anti-cancer effects on liver tumors and the underlying mechanisms have not been identified. Herein, employing flow cytometry, and Western blot analysis, we demonstrate that RY10-4 can induce cell cycle arrest, intracellular reactive oxygen species (ROS) production and apoptosis in HepG2 cells. In HepG2 cell xenograft tumor model, RY10-4 significantly inhibited the growth of tumors and induced apoptosis in tumor cells, with little side effects. Moreover, RY10-4 caused the suppression of STAT3 activation, which may be involved the apoptosis induction. In addition, RY10-4 inhibited the proliferation of Hep3B and HuH-7 human hepatocellular carcinoma cells in a concentration-dependent manner. Taken together, our results suggest that RY10-4 has a great potential to develop as chemotherapeutic agent for liver cancer.

## Introduction

According to the latest global cancer statistic, hepatocellular carcinoma is the third highest cause of cancer-related death worldwide [1]. As one of the major cancer treatment modalities, chemotherapy has effectiveness in prolonging and improving the quality of life for the patients [2]. Research of small molecular compounds with anti-tumor activity has great benefits for developing new chemotherapy agents. Some small molecular compounds, such as piperlongumine [3] and obtusaquinone [4], targeting and selectively killing cancer cells, could be promising cancer therapeutic agents.

Utilizing natural products as lead compound is a useful and practicable method in drug development. Based on the structure of a natural product protoapigenone, RY10-4 was designed and synthesized by Yuan *et al*. in our laboratory, and it showed potent anti-tumor activities against multiple human cancer cell lines [5]. Its chemical structure was close to protoapigenone, while its anti-tumor activity was improved and side effects were reduced. The mechanisms mediating the anti-tumor activity of RY10-4 included induction of autophagy or apoptosis in human breast or lung cancer cell lines, respectively [6,7]. In a previous screening study, RY10-4 showed remarkable anti-proliferative effects on HepG2 human hepatocellular cancer cells *in vitro*, and inhibited the growth of mouse hepatocellular H22 tumor *in vivo* [5]. However, further research is needed to fully understand the anti-tumor effects of RY10-4 in liver cancer and its potential mode of action. Here, we demonstrate that RY10-4induces apoptosis in HepG2 liver cancer cells both *in vitro* and *in vivo*, and thereby has ability to suppress liver tumors.

## Materials and Methods

### Regents and antibodies

RY10-4 (>95%) was prepared previously in our laboratory as described by Yuan et al. [5]. Sulforhodamine B (SRB) was purchased from J&K Scientific (Beijing, China). Dimethyl sulfoxide (DMSO) and N-acetylcysteine (NAC) were purchased from Sigma Aldrich (St. Louis, MO, USA). Hoechst 33342, propidium iodide (PI), reactive oxygen species assay kit (DCFH-DA), RIPA lysis buffer, BCA protein assay kit, and BeyoECL plus chemiluminescence kit were obtained from Beyotime Inc. (Shanghai, China). Annexin V-FITC/PI apoptosis detection kits was purchased from KeyGEN BioTECH (Nanjing, China). Specific antibodies against Bcl-2, Bax, p53, cyclin E, CDK2, cleaved caspase3, actin, STAT3, p-STAT3, p21, GAPDH and corresponding secondary antibodies were obtained from Cell Signaling Technology (Beverly, MA, USA).

### Cell line and cell culture

Human hepatocellular cancer HepG2, Hep3B and HuH-7 cell lines were purchased from China Center for Type Culture Collection, CCTCC). Cells were cultured in Dulbecco’s modified Eagle’s medium (DMEM, Hyclone) supplemented with 10% (v/v) fetal bovine serum (FBS, Hyclone) and antibiotic solution (100 μg/ml streptomycin and 100 IU/ml penicillin), in a humidified atmosphere of 5% CO_2_ at 37°C.

### SRB assay

The cell viability was measured by SRB assay as described previously [6]. In brief, HepG2 cells were seeded in 96-well culture plates (5000 cells per well), and treated with a series of concentrations of RY10-4. After the treatment, cells were fixed with 10% trichloroacetic acid for 1 h at 4°C, and washed with 1% acetic acid. The fixed cells were stained with 0.4% SRB for 15 min, washed four times and left to dry at room temperature. The bound dye was solubilized with Tris-Base solution, and the absorbance at 540 nm was recorded using a microplate reader (BioTek Instruments, Inc. USA). To explore the role of ROS in RY10-4-induced cell death, HepG2 cells were pretreated with the ROS scavenger NAC (5 mM) for 2 h followed by the treatment with RY10-4 and the cell viability was measured.

### Clonogenic assay

HepG2 cells in the exponential phase of growth were seeded in 6-well plates (1000 cells per well), and allowed to adhere. Following treatment with serial concentrations of RY10-4 for 24 h, the cells were cultured in fresh medium without drugs until visible colonies formed. The cell colonies were then fixed with 4% paraformaldehyde and stained with 0.5% crystal violet staining solution.

### Cell cycle analysis

For cell cycle analysis, the phase distribution of DNA content was determined using PI staining. After treatment with 0, 0.9, 1.8, and 2.7μM of RY10-4 for 24 h, HepG2 cells were harvested and fixed by 70% ethanol overnight at -20°C. Then the cells were washed and stained with PI staining solution (50 μg/ml PI and 10 μg/ml RNase) for 30 min in the dark. The cell cycle distribution was analyzed by flow cytometry using Cell-Quest software (Becton Dickinson, USA).

### Measurement of intracellular ROS production

DCFH-DA was used as a fluorescent probe to measure intracellular ROS production by flow cytometry. Briefly, HepG2 cells were seeded into 6-well plates. After adhesion, the cells were treated with varying concentrations of RY10-4. Then the medium was removed and the cells were incubated with 10 μM DCFH-DA for 20 min in the dark. The stained cells were collected and analyzed by flow cytometry (Becton Dickinson, USA). In some experiments, the cells were pretreated with the ROS scavenger NAC.

### Apoptosis detection

HepG2 cells were treated with varying concentrations of RY10-4 and analyzed for apoptosis using either Hoechst 33342 staining or Annexin V-FITC/PI. In brief, after the treatment, cells were stained with Hoechst 33342 for 10 min at room temperature, then washed and observed under a fluorescent inverted microscope (Nikon Eclipse, Japan). Alternatively, cells were collected and stained with Annexin V-FITC/PI apoptosis detection kit according to the manufacturer’s protocol; the apoptotic cells were detected by flow cytomety (Becton Dickinson, USA).

### Western blot assay

Protein expression was determined by Western blot. Briefly, the total proteins were extracted by RIPA lysis buffer containing 1% phenylmethanesulfonylfluoride (PMSF) and 1% cocktail. The proteins were separated by SDS-PAGE and transferred onto a PVDF membrane (Bio-Rad). The membrane was blocked by 5% nonfat milk for 1 h at room temperature and incubated with a specific primary antibody overnight at 4°C. The membrane was then washed and incubated with a corresponding secondary antibody for 2 h at room temperature. The specific protein-antibody complex was visualized by ECL plus chemiluminescence kit.

### Nude mice xenograft model

All animal experiments were approved by the Institutional Animal Ethical Committee of Huazhong University of Science and Technology, China. All experimental animals received care in compliance with the guidelines proposed by Institutional Animal Care and Use Committee, and all efforts were made to minimize suffering. Four- to five-week-old BALB/c nude mice (No. 4304701485) were purchased from Hunan SJA Laboratory Animal Co. Ltd. (Hunan, China). The mice were housed under specific pathogen-free conditions with free access to rodent chow and water at 20–22°C with 12-hour light-dark cycle, and acclimatized for one week before the experiment. Then HepG2 cells (2×10^6^) were injected subcutaneously into each mouse. When the volumes of established tumors reached 100–200 mm^3^, the mice were randomized into the following groups: saline control, low-dose RY10-4 (5 mg/kg) treated group, and high-dose RY10-4 (50 mg/kg) treated group. Body weight and tumor volume of the animals were measured once a week. The mice were observed daily for ulceration, abdominal swelling, emaciation and/or other signs of distress. When a mouse presented the above symptom(s) and it was believed that the mouse would not survive, it was sacrificed and the time of death was recorded. Tumor volume was calculated using the formula: V = *ab*^2^/2 (*a*, *b* are tumor length and width, respectively). The relative tumor volume (RTV) = V_t_/V_0_, where V_0_ is the tumor volume measured at the time of the first drug administration and V_t_ represents each tumor measurement after the treatment. At the experimental endpoint, all mice were sacrificed by cervical dislocation under ether anesthesia.

### Histopathology and immunohistochemistry analysis

One part of the tumor tissue sample was fixed with 4% paraformaldehyde and embedded in paraffin. Tissue sections (4 mm) were prepared and stained with hematoxylin/eosin (H&E) according to the standard protocol. The other part of the sample was frozen at -80°C, then frozen-sectioned into 4 to 10 μm thick sections. The sections were blocked with 5% BSA and incubated with primary antibodies at 4°C overnight. After washing, the sections were incubated with HRP-conjugated secondary antibody, followed by applying a DAB color development kit (Beyotime Inc., China). The images were captured under a microscope (Nikon, Japan).

### Statistical analysis

Data were expressed as means ± S.D. from at least three independent experiments. Statistical analysis was performed using one-way ANOVA with Dunnett’s posttest. *P* values were calculated using Student’s *t* test (alpha level: 0.05, two-tailed). The differences between the groups were considered significant at *P* values less than 0.05.

## Results

### The anti-proliferative effects of RY10-4 on HepG2 cells

**Fig 1:**
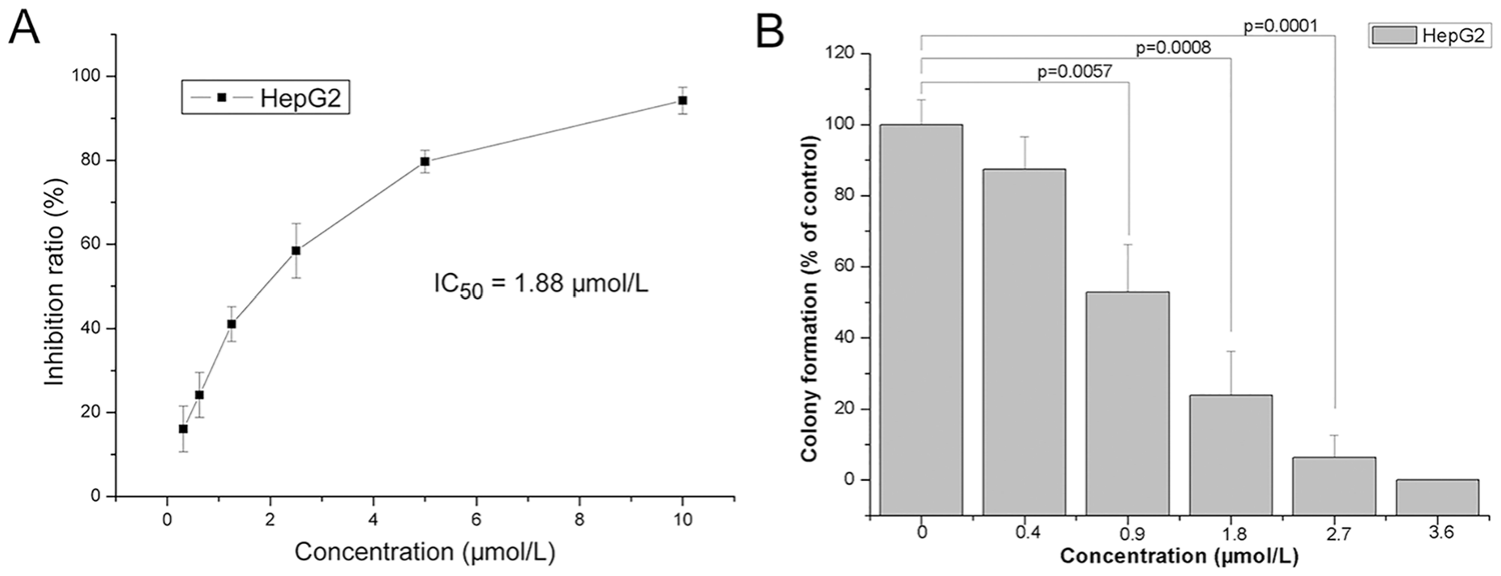
The anti-proliferative effects of RY10-4 on HepG2 cells. (A) HepG2 cells were treated with different concentrations (0.312, 0.625, 1.25, 2.5, 5, and 10 μM) of RY10-4; cell growth inhibition ratio was determined by SRB assay, and the IC_50_ value was 1.88 μM. (B) HepG2 cells were seeded in a very low density, and treated with RY10-4 for 24 h. Then cells were grown in fresh medium without drugs to form colonies. The colony formation ratio (% of control) was calculated.

When results of the SRB assay are linear over a range of cell numbers, the assay can be used to determine drug-induced cytotoxicity [8]. As shown in Fig 1A, RY10-4 inhibited the proliferation of HepG2 cells in a concentration-dependent manner. The half maximal inhibitory concentration (IC_50_) value of RY10-4 on HepG2 cells was 1.88 μM. In addition, the clonogenic assay showed that RY10-4 treatment significantly decreased the colony numbers of HepG2 cells compared with control group. The colony formation of HepG2 cells was almost completely abolished by RY10-4 at the concentration of 3.6 μM (Fig 1B).

### RY10-4 induced cell cycle arrest in HepG2 cells

**Fig 2:**
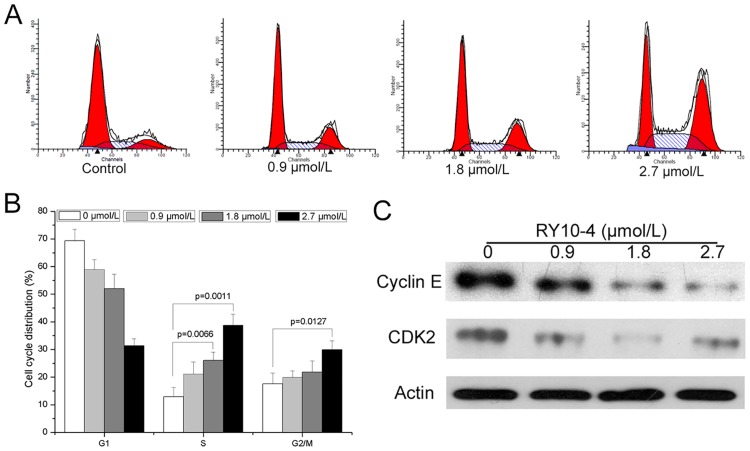
The effect of RY10-4 on cell cycle distribution of HepG2 cells. (A) Cells were treated with 0, 0.9, 1.8, and 2.7μM RY10-4 for 24 h, and the DNA content was analyzed using propidium iodide staining and flow cytometry. (B) Cell cycle distributions, presented as mean ± SD of three independent experiments. (C) After the treatment with RY10-4, expression of cell cycle-related proteins cyclin E and CDK2 was analyzed by Western blot.

The cell cycle distribution of HepG2 cells after RY10-4 treatment was assessed by analysis of DNA content using PI staining. As shown in Fig 2A and 2B, RY10-4 at concentrations of 0.9, 1.8, and 2.7 μM induced a significant increase in the percentage of cells in S phase. Also 2.7 μM RY10-4 induced a remarkable increase in G2/M phase (*P* = 0.0127). Western blot analysis showed that the expression of cell cycle-related proteins cyclin E and CDK2 in HepG2 cells was decreased by RY10-4 treatment (Fig 2C).

### RY10-4 induced intracellular ROS production in HepG2 cells

**Fig 3:**
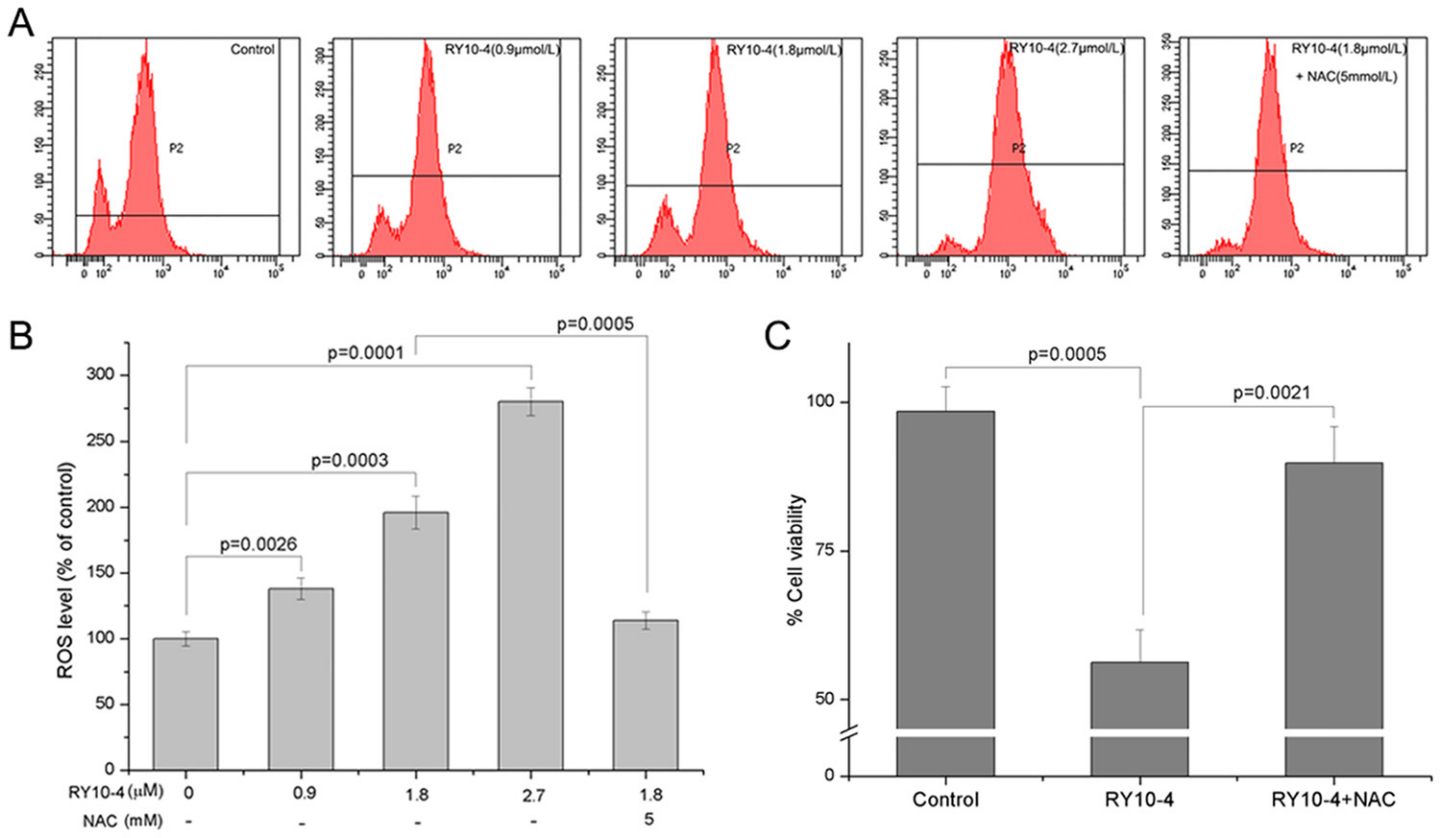
The effect of RY10-4 on intracellular ROS production. (A) HepG2 cells were treated with indicated concentrations of RY10-4, and the intracellular ROS production was detected as described in Methods. (B) The intracellular ROS levels (% of control), presented as mean ± SD of three independent experiments. (C) HepG2 cells were treated with RY10-4 in the absence or presence of NAC pretreatment; the cell viability was determined by SRB assay.

The intracellular ROS production was detected using oxidation sensitive fluorescent probe DCFH-DA. As shown in Fig 3A, after treatment with indicated concentrations of RY10-4, the intracellular ROS production was dramatically increased in HepG2 cells. A concentration-dependent manner was observed, and pre-treatment with NAC (5 mM) for 2 h almost completely reversed the RY10-4-induced ROS production in HepG2 cells (Fig 3B). Consistently, pre-treatment with NAC also prevented the cell viability decrease induced by RY10-4 (Fig 3C).

### RY10-4 induced apoptosis in HepG2 cells

**Fig 4:**
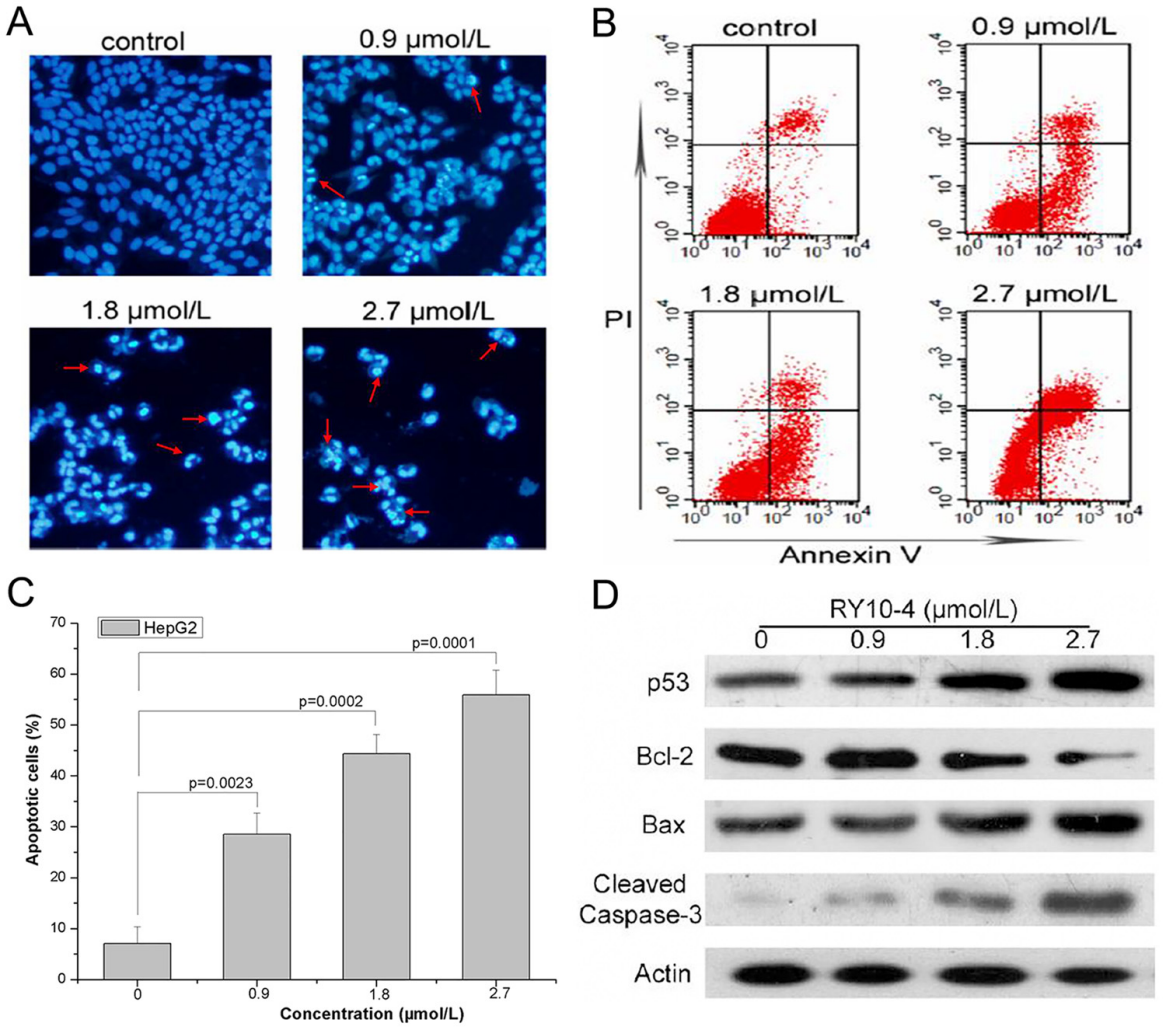
RY10-4 induced apoptosis in HepG2 cells. (A) Cells were treated with indicated concentrations of RY10-4 for 24 h, and the nuclei were stained by Hoechst 33342. Arrows indicate condensed and fragmented nuclei. (B) Flow cytometry assay to detect apoptosis in HepG2 cells using Annexin V/PI staining. Representative flow cytometry profiles are shown. (C) The percentage of apoptotic cells, presented as mean ± SD of three independent experiments. (D) Expression of apoptosis-related proteins was analyzed by Western blot in HepG2 cells untreated or treated with RY10-4.

The fluorescence staining of nuclei and Annexin V-FITC/PI staining were performed to detect apoptosis in HepG2 cells. Fig 4A demonstrated that after treatment with indicated concentrations of RY10-4, condensed nuclei and apoptotic bodies were observed in HepG2 cells by staining with Hoechst 33342. Annexin V-FITC/PI staining also showed that RY10-4 induced apoptosis in a concentration-dependent manner (Fig 4B). Comparing with the control group, the percentage of apoptotic cells was significantly increased in RY10-4-treated groups (Fig 4C). In addition, apoptosis-related proteins p53, Bcl-2, Bax, and cleaved caspase-3 were analyzed by Western blot. As shown in Fig 4D, the expression of anti-apoptotic protein Bcl-2 decreased after the treatment with RY10-4, while the expression of p53, Bax and cleaved caspase-3 increased in a concentration-dependent manner. Taken together, these results suggest that RY10-4 can induce apoptosis in HepG2 cells.

### Anti-tumor activity of RY10-4 in HepG2 cell xenograft model

**Fig 5:**
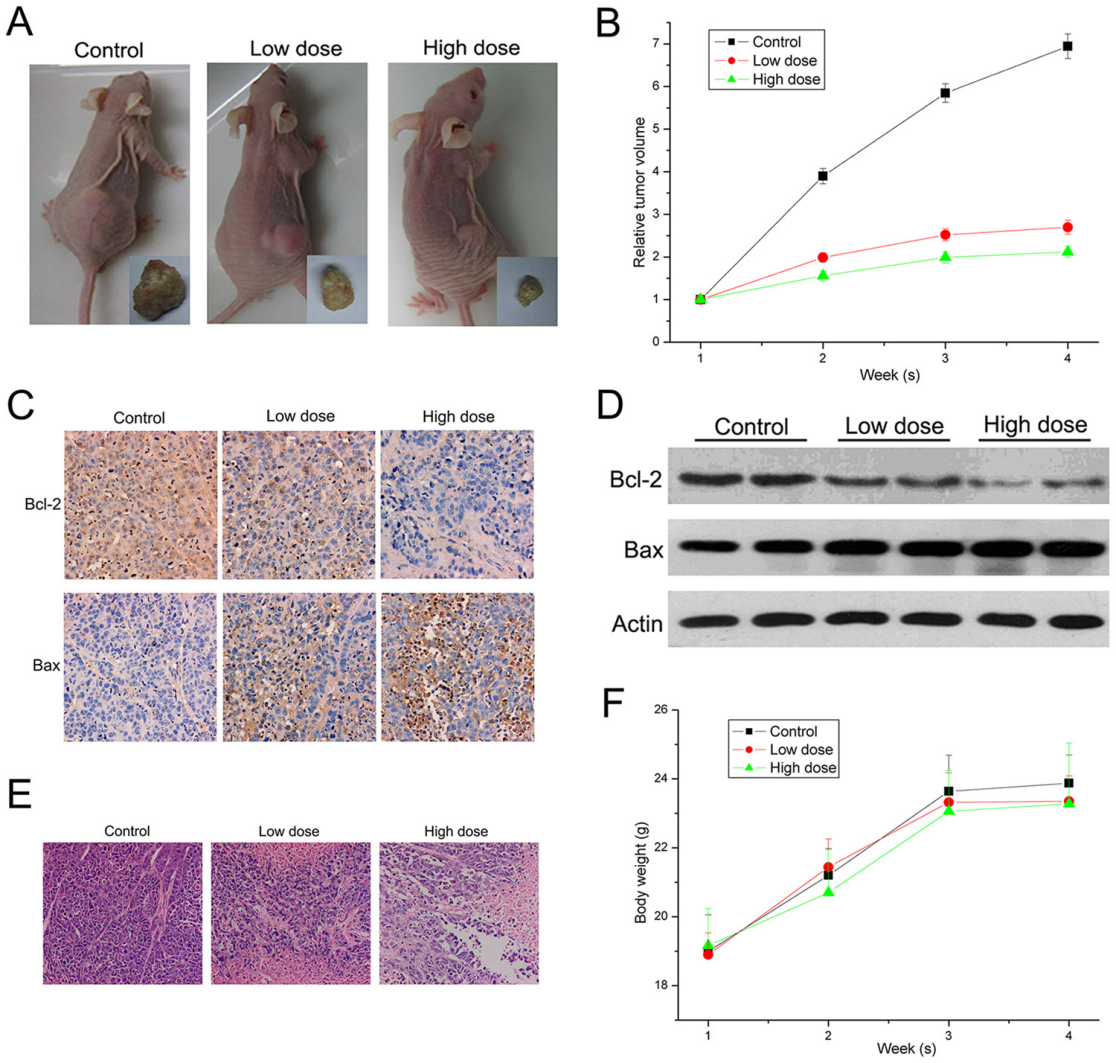
Anti-tumor activity of RY10-4 in HepG2 cell xenograft model. (A) Representative xenograft tumors obtained from control mice and mice treated with RY10-4. (B) The relative tumor volume (RTV) was calculated according to the formula in Methods. (C and D) Expression of Bcl-2 and Bax in tumor tissue was analyzed by immunofluorescence staining and Western blot. (E) HE staining was performed for pathological examination of tumor tissue in different groups. (F) The average body weights of mice in each group were evaluated.

The anti-tumor effect of RY10-4 *in vivo* was evaluated using a nude mouse xenograft model. All mice survived throughout the whole experiment period, and they were sacrificed under ether anesthesia at the experimental endpoint. As shown in Fig 5A, treatment with RY10-4 resulted in remarkable growth inhibition of HepG2 cell xenograft tumors compared with saline-treated control group. At the end of the experiment, tumor volume was 1326 ± 407 mm^3^, 530 ± 201 mm^3^ and 411 ± 108 mm^3^ in the control group, low-dose RY10-4 treatment group and high-dose RY10-4 treatment group, respectively. The average tumor volume in the control group reached nearly seven-fold of the initial volume, while the tumors in the treatment groups were increased only about two-fold, as compared to the initial volume (Fig 5B). We also analyzed the expression of apoptosis-related proteins Bcl-2 and Bax in tumor tissue samples from each group by immunohistochemistry and Western blot. As shown in Fig 5C and 5D, compared with the control group, expression of the anti-apoptotic protein Bcl-2 decreased while the pro-apoptotic protein Bax increased both at low and high doses of RY10-4. The HE staining also showed large areas of tumor cell death in the treatment groups (Fig 5E). These results suggested that RY10-4 could induce hepatocellular tumor apoptosis *in vivo*. The body weight in each group did not change significantly throughout the experiment (Fig 5F), and RY10-4 treatment did not affect blood biochemical indexes of the tumor-bearing nude mice (S1 Table), suggesting that RY10-4 probably has little side effects *in vivo*.

### The molecular mechanisms of apoptosis induction by RY10-4 in HepG2 cells

**Fig 6:**
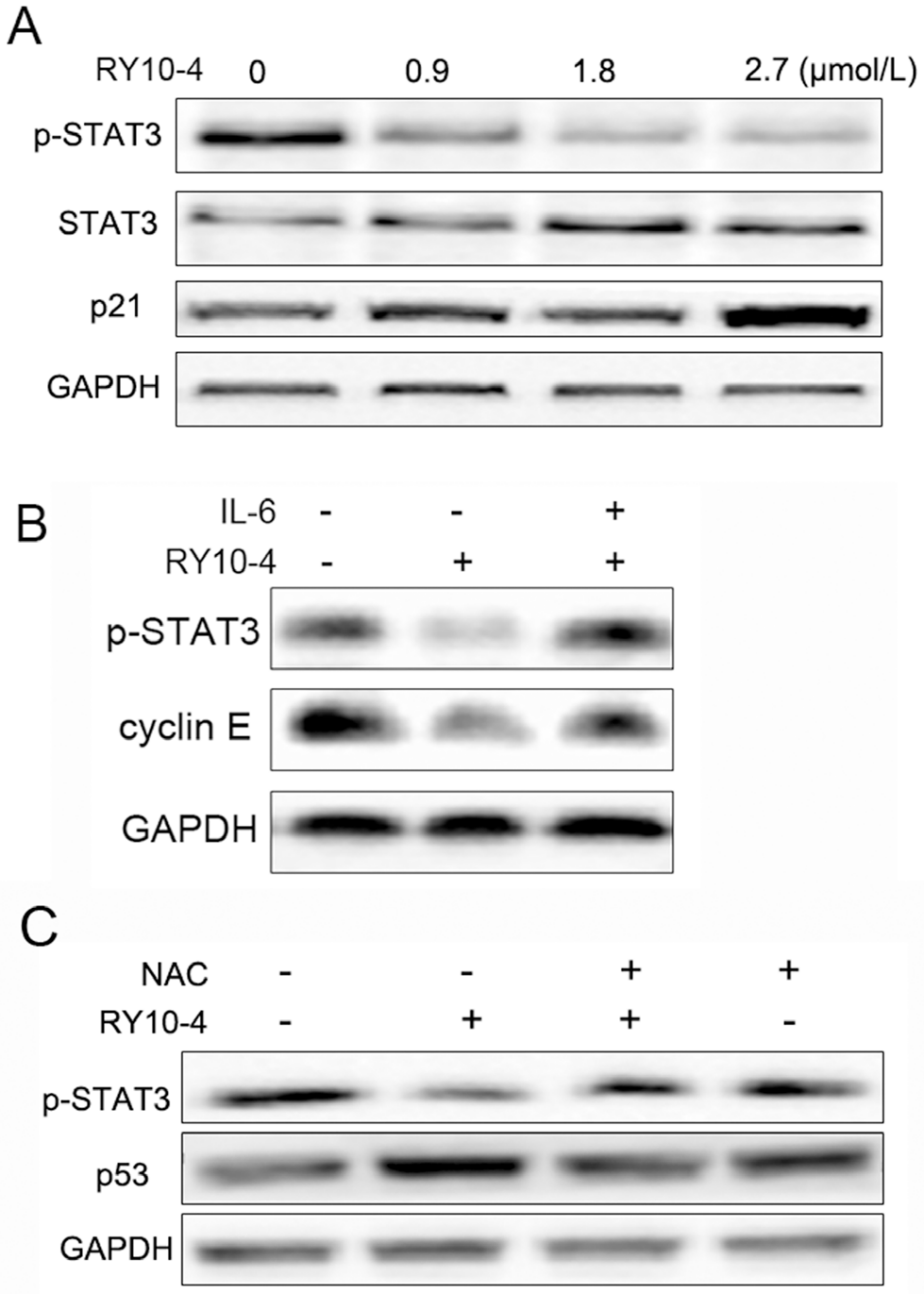
The molecular mechanisms of apoptosis induction by RY10-4 in HepG2 cells. (A) HepG2 cells were treated with indicated concentrations of RY10-4, and the expression of p-STAT3 and p21 was analyzed by Western blot. (B) HepG2 cells were treated with RY10-4 with or without IL-6 stimulation; expression of p-STAT3 and cyclin E was analyzed by Western blot. (C) HepG2 cells were treated with RY10-4 in the absence or presence of NAC pretreatment. The expression of p-STAT3 and p53 was analyzed by Western blot.

Since STAT3 regulates the expression of various tumor growth related oncogenic genes and plays a key role in cell proliferation [9], we investigated whether RY10-4 can regulate constitutive STAT3 activation in HepG2 cells. As shown in Fig 6A, RY10-4 inhibited constitutive phosphorylation of STAT3 (Tyrosine 705) in HepG2 cells. RY10-4 up-regulated the expression of p21, which may be involved RY10-4-induced cell cycle arrest. In addition, IL-6 (10 ng/ml) reversed RY10-4-induced STAT3 inactivation, followed by the increase of cyclin E expression in RY10-4-treated HepG2 cells (Fig 6B). Moreover, pre-treatment with NAC inhibited STAT3 abrogation and p53 increase caused by RY10-4 in HepG2 cells (Fig 6C).

### The anti-proliferative effects of RY10-4 on other hepatocellular carcinoma cell lines

**Fig 7:**
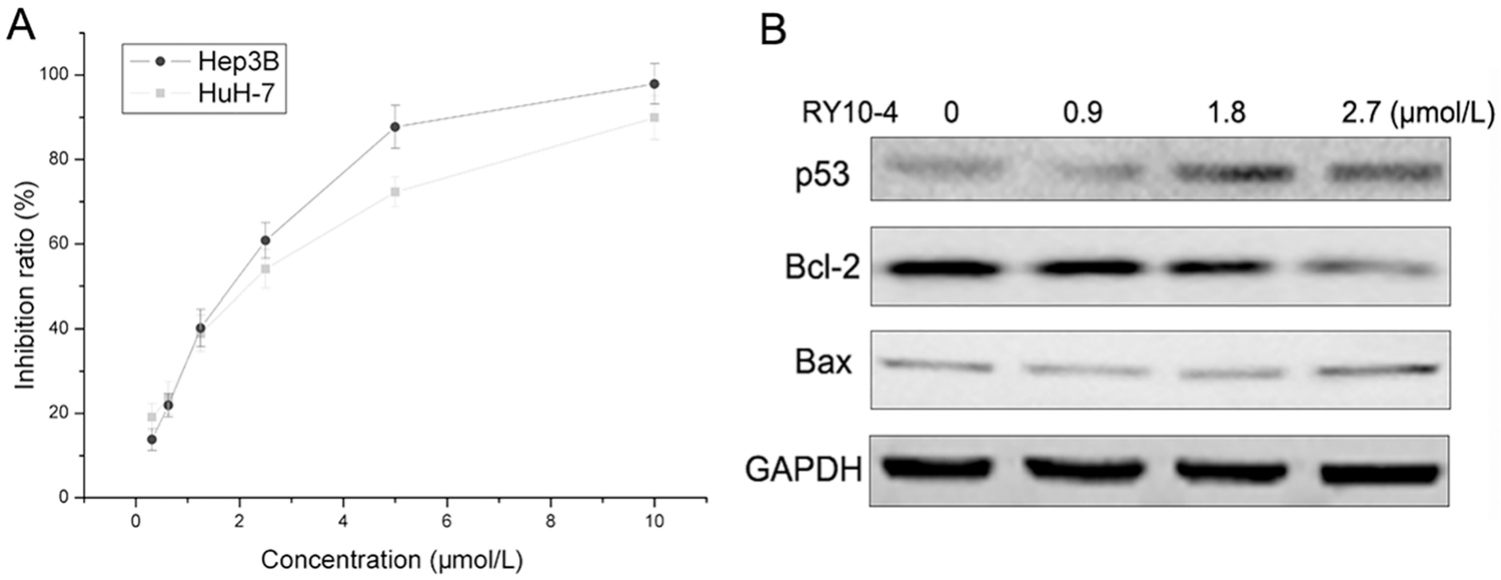
The anti-proliferative effects of RY10-4 on other hepatocellular carcinoma cells. (A) Hep3B or HuH-7 cells were treated with series concentrations of RY10-4, and cell growth inhibition ratio was determined by SRB assay. (B) Hep3B cells were treated with RY10-4, and the expressions of apoptosis-related proteins p53, Bcl-2 and Bax were analyzed by Western blot.

To observe the anti-proliferative effects of RY10-4 against other human liver cancer cell lines, we choose Hep3B and HuH-7 hepatocellular carcinoma cell lines. As shown in Fig 7A, RY10-4 inhibited the proliferation of Hep3B and HuH-7 cells in a concentration-dependent manner, and Hep3B cells were more sensitive. Next, we detected the expressions of apoptosis-related proteins in RY10-4-treated Hep3B cells. As shown in Fig 7B, after treatment with RY10-4, the expression of p53 and Bax increased, while the expression of Bcl-2 decreased.

## Discussion

The natural product protoapigenone and several of its derivatives have shown potent anti-tumor effects against certain of human cancer cell lines [10,11]. Protoapigenone could be considered as a useful lead compound to discover new chemotherapy agents. In our laboratory, RY10-4 was designed and synthesized as a protoapigenone analog, as an attempt to develop a novel anti-tumor compound. According to some previously researches and reports [12,13], RY10-4 had potent anti-tumor activities against human breast cancer cells. In another report, RY10-4 could induce autophagic cell death in MCF-7 cells while protoapigenone could not [6]. In addition, Xue *et al*. reported that RY10-4 induces apoptosis and inhibits invasion in human lung cancer A549 cells by inhibiting STAT3 signaling [7]. These studies indicate that RY10-4 has great potential as a novel anti-tumor agent.

In the present study, we evaluated the anti-tumor activity of RY10-4 in liver cancer. The results showed that RY10-4 effectively inhibited proliferation of HepG2 liver cancer cells *in vitro* with the IC_50_ value of 1.88 μM. Furthermore, the clonogenic assay was performed to determine the long-term effects of RY10-4 on HepG2 cells. The clonogenic assay correlates well with the assay of tumorigenicity *in vivo*, and it has proven predictive value in chemosensitivity test of antitumor agents [14]. Our results indicated a concentration-dependent inhibition in clonogenicity in RY10-4-treated HepG2 cells (Fig 1B), suggesting that RY10-4 has a curative potential against the hepatocellular cancer.

Induction of cell cycle arrest and apoptosis are the core mechanisms of action of many anti-tumor agents. It has been reported that protoapigenone and its analogs as well as RY10-4 could cause cell cycle arrest and apoptosis in some human cancer cell lines [13,15–17]. Here, we analyzed cell cycle distribution and apoptosis of HepG2 cells after treatment with RY10-4. We found that RY10-4 could induce cell cycle arrest in S and G2/M phases, which might be attributed to the down-regulation of the cell cycle-related proteins cyclin E and CDK2 (Fig 2). In addition, Hoechst 33342 staining and Annexin V-FITC/PI staining showed that RY10-4 also induced concentration-dependent apoptosis in HepG2 cells (Fig 3). P53 is one of the most important tumor suppressors and targeting p53 family proteins may prove therapeutically useful [18,19]. P53 can be stimulated by hypoxia, carcinogens, oxidative stress and other stressors, inducing cell cycle arrest or apoptosis *via* several pathways [20]. Bcl-2 family proteins, including pro- and anti-apoptotic regulators, also play an important role in apoptosis induction and cell survival [21,22]. In addition, the caspase family proteins like caspase-3 are executors of apoptosis and activation of caspases will initiate apoptosis [23]. After treatment with RY10-4, the expression of p53, Bax and cleaved caspase-3 increased, while the expression of Bcl-2 decreased in HepG2 cells (Fig 3D). These results indicate that induction of apoptosis may be the underlying mechanism of anti-tumor activity for RY10-4 against human hepatocellular cancer HepG2 cells.

Compelling evidences suggest that ROS are implicated in a variety of cellular programs and plays a key role in human physiological and pathological processes [24]. Cancer cells usually exhibit higher basal levels of ROS, having a different redox status comparing with normal cells, and high ROS levels can often damage the cells [25,26]. Therefore inducing ROS production in cancer cells can have a positive therapeutic impact. Compounds like piperlongumine, obtusaquinone and psoralidin induce ROS production thereby inhibiting proliferation of human cancer cells [3,4,27]. In this study, we demonstrated that RY10-4 induced intracellular ROS production, which could be reversed by pretreatment with NAC in HepG2 cells. Also RY10-4-induced cell death was partially prevented by pretreatment NAC (Fig 4). These results suggest that ROS production may play an important role in RY10-4-induced HepG2 cell death. The relationship between RY10-4-induced apoptosis and ROS production will be further investigated in a follow-up study. Numerous evidences indicate that STAT3 plays a central role in the development and progression of many human tumors, and suppression of STAT3 activation leads to growth inhibition and apoptosis in tumor cell lines as well as mouse xenograft models [9,28]. In our study, we found that RY10-4 suppressed STAT3 activation in HepG2 cells, and the effect was dependent on ROS generation (Fig 6). Taken together, ROS dependent inactivation of STAT3 may be the molecular mechanism of RY10-4 for its anti-liver tumor activity.

Generally speaking, only clinical trials can ultimately determine whether a new agent is effective for cancer therapy in patients. However there are many ethical, medical and economic limitations and constraints for clinical trials, hence a convenient experimental system is of great importance for anticancer drug screening [29]. The nude mice xenograft model is normally used to evaluate the biological activity of anticancer agents *in vivo* [30,31]. Human cancer cells can grow as xenografts in an immunodeficient mouse. To some degree, this model is helpful to predict clinical response to drug therapy. Here, we established the HepG2 cell xenograft model to evaluate the antitumor activity of RY10-4. We found that RY10-4 induced apoptosis on tumor and significantly inhibited its growth *in vivo* (Fig 5). In addition, RY10-4 probably has only minor side effects *in vivo*, as it did not affect animal body weight and blood biochemical indexes.

In conclusion, this paper demonstrates that RY10-4 can remarkably inhibit proliferation of human hepatocellular cancer HepG2 cells both *in vitro* and *in vivo*. Induction of apoptosis may be the underlying mechanism for its anti-tumor activity. RY10-4 suppressed liver tumor growth *in vivo* without significant hematological toxicity, hepatotoxicity or nephrotoxicity. In addition, RY10-4 inhibited the proliferation of Hep3B and HuH-7 human hepatocellular carcinoma cells in a concentration-dependent manner. These results suggest that RY10-4 has great potential as a novel chemotherapeutic agent for liver cancer.

## Supporting Information

S1 Table Complete blood count and biochemical profile for the nude mice after treatment with RY10-4 for 4 weeks (mean ± SD, n = 8). (DOCX)
