# Supplementary material for: RY10-4 Inhibits the Proliferation of Human Hepatocellular Cancer HepG2 Cells by Inducing Apoptosis In Vitro and In Vivo
Source: PLoS One. 2016 Mar 14;11(3):e0151679. doi: 10.1371/journal.pone.0151679 (PMC4790938; doi:10.1371/journal.pone.0151679)
Supplement: S1 Table — (DOCX) [file pone.0151679.s001.docx]

**S1 Table. Complete blood count and biochemical profile for the nude mice after treatment with RY10-4 for 4 weeks (mean ± SD, n=8)**

| Groups | Control | Low dose | High dose |
| --- | --- | --- | --- |
| WBC（×10^9^/L） | 12.80±1.13 | 11.10±1.27 | 11.45±0.62 |
| RBC（×10^12^/L） | 11.15±0.64 | 9.92±0.85 | 10.49±1.52 |
| Hb（g/L） | 154±19.80 | 152±7.07 | 151±21.21 |
| MCV（fL） | 50.70±0.28 | 50.25±0.49 | 50.60±0.99 |
| ALT（U/L） | 78.0±7.1 | 73.0±12.7 | 69.5±9.2 |
| AST（U/L） | 284.5±30.4 | 277.0±69.3 | 253.5±24.7 |
| BUN（mmol/L） | 11.00±1.60 | 10.69±0.58 | 11.20±0.90 |
| CREA（μmol/L） | 5.45±1.34 | 5.05±2.33 | 5.35±0.78 |
